# The GLP-1 analogue, exendin-4, improves bone material properties and strength through a central relay in ovariectomized mice

**DOI:** 10.1152/ajpendo.00086.2025

**Published:** 2025-08-11

**Authors:** Morgane Mermet, Jessica Denom, Aleksandra Mieczkowska, Méline Wery, Emma Biggs, Fiona M. Gribble, Frank Reimann, Christophe Magnan, Celine Cruciani-Guglielmacci, Guillaume Mabilleau

**Affiliations:** 1 Univ Angers, Nantes Université, ONIRIS, Inserm, RMeS, UMR 1229, SFR ICAT, F-49000, Angers, France; 2 Université Paris Cité, CNRS, BFA, UMR 8251, F-75205, Paris, France; 3 Univ Angers, SFR ICAT, F-49000 Angers, France; 4 Institute of Metabolic Science & MRC Metabolic Diseases Unit, University of Cambridge, Cambridge, UK; 5 CHU Angers, Departement de Pathologie Cellulaire et Tissulaire, UF de Pathologie osseuse, 49933 Angers, France

**Keywords:** GLP-1, exendin-4, intracerebroventricular administration, bone material properties, bone quality

## Abstract

Glucagon-like peptide-1 (GLP-1) has previously been shown to be indispensable for optimal bone strength by acting at the bone material level. However, it was not fully clear whether the effects of GLP-1 were mediated by direct or indirect actions on bone cells. In the present study, we were unable to demonstrate the expression of the GLP-1 receptor (GLP-1r) in bone tissue at the gene expression level using qPCR and in situ hybridization, or at the protein level. Furthermore, the peripheral administration of exendin-4, a specific GLP-1r agonist, in ovariectomized (OVX) BALB/c mice enhanced post-yield displacement (18%) and energy-to-fracture (24%), as well as bone volume/total volume (BV/TV) (11%), trabecular number (Tb.N) (6%), and collagen maturity (18%). These bone effects were still observed when exendin-4 was centrally administered into the lateral cerebral ventricle. On the other hand, the peripheral administration of exendin-4 coupled to bovine serum albumin, a GLP-1r agonist that cannot penetrate the brain, failed to replicate the positive effects on bone despite increased calcitonin secretion. Altogether, these data confirm that GLP-1r agonists represent an interesting approach for managing bone fragility due to ovariectomy, but also suggest that GLP-1r agonists require a central relay—yet to be identified—to exert positive effects on bone physiology. Further studies are needed to decipher the mechanisms of action of GLP-1 and GLP-1r agonists on bone physiology.

**Figure:**
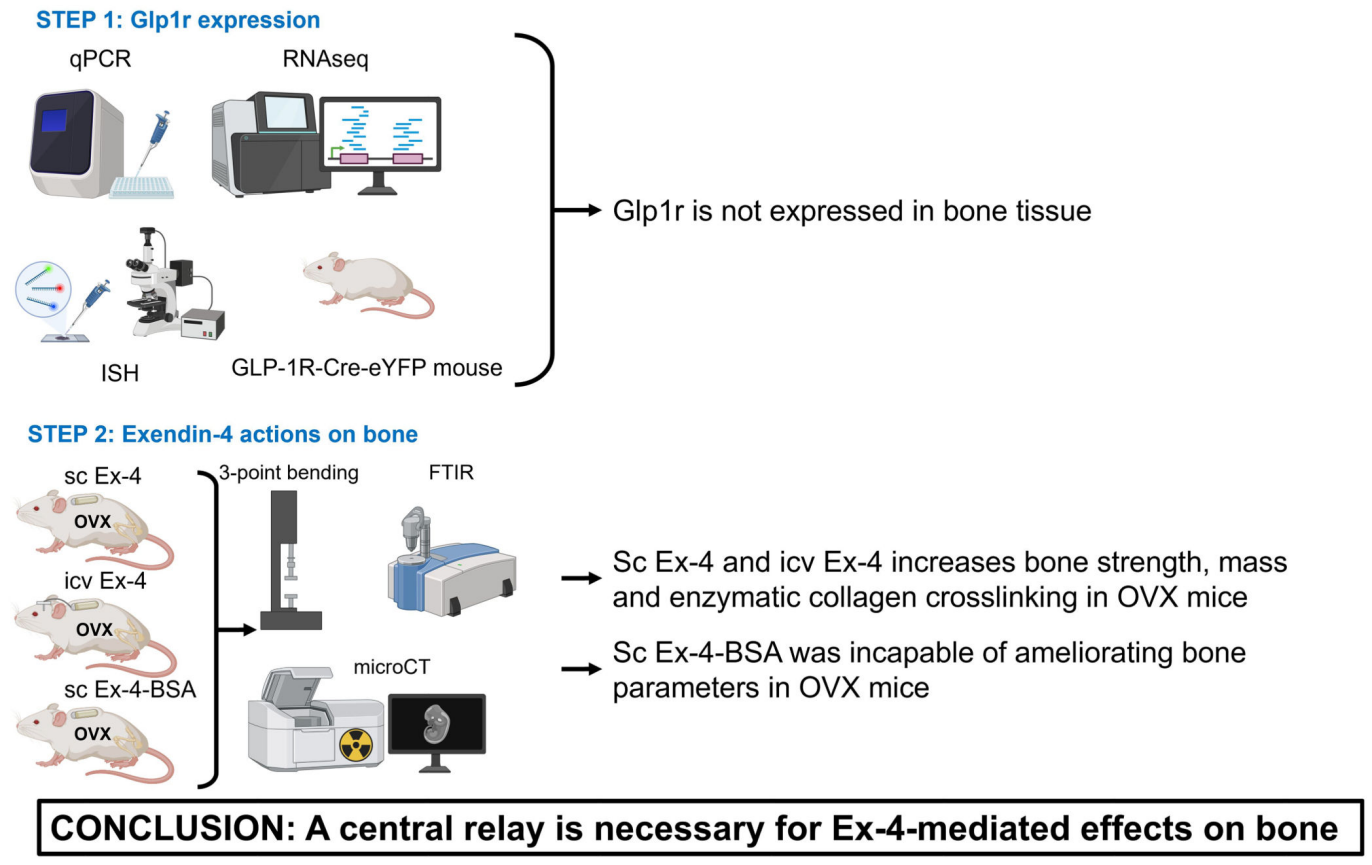

## 1 Introduction

Due to ageing of the population, the occurrence of bone fragility and fracture has risen significantly worldwide and will continue in the future. In 2019 in the European Union only, the total direct cost of fragility fractures amounted to €56.9 billion, a 64% increase compared with the figure in 2010 (1). Bone fragility is characterized by reduced bone mass, disruption of bone microarchitecture and deterioration of the quality of the bone material itself. Although approved anti-osteoporotic drugs increase bone mineral density, the risk of bone fracture is only reduced by 30-40% in hip and long bones in treated individuals (1–4). This suggests that factors beyond bone mass are important for an optimum bone strength.

Previously, we and others have highlighted the beneficial role of several gut hormones on bone physiology (5). Among gut hormones, glucagon-like peptide-1 (GLP-1) was reported to be beneficial for bone strength. Indeed, animals with a genetic impairment of the GLP-1 receptor (GLP-1r) presented with reduced bone mass, deterioration of bone microarchitecture and alterations of bone material properties, especially enzymatic collagen crosslinking, that jeopardized bone strength (6, 7). In humans, GLP-1r gene polymorphisms have also been reported to impact bone mineral density and mass (8) and exogenous GLP-1 administration rapidly modulates bone remodeling (9–12). Due to the important role of GLP-1 on glucose and energy metabolism and its short half-life in the circulation, several degradation-resistant GLP-1r agonists have been developed and approved for the treatment of type 2 diabetes and more recently obesity (13). These GLP-1r agonists, administered subcutaneously, have proved useful in several animal models of bone fragility to enhance bone strength (14–20). In meta-analysis of randomized clinical trials, GLP-1r agonists were reported with a neutral or slightly beneficial effects on reducing the incidence of bone fracture (21), although more recent data suggest that liraglutide and lixisenatide contribute to reduce fracture in individuals affected by type 2 diabetes mellitus (22).

However, the exact mechanism of action of GLP-1r agonist in improving bone strength remains to be elucidated. Indeed, it is not clear whether improvement of bone strength resulted from activation of skeletal GLP-1r or whether it requires extra-skeletal receptors as reported for the GIP/GIP receptor pathway (23). Furthermore, the unambiguous presence of a functional GLP-1r in bone cells remains up to now controversial due to poorly characterized cell lines, non-selective use of PCR primers or lack of sufficiently selective and commercially available antibodies (24, 25). Recently, despite clear effects of GLP-1 and its analogues on improving enzymatic collagen crosslinking upon peripheral administration in animals, Mieczkowska et al. were incapable of inducing a change in collagen crosslinking in vitro in osteoblast cultures (24).

The main objectives of this study were to better understand how GLP-1 controls bone strength in the ovariectomy-induced bone fragility model and to decipher whether a functional GLP-1r is expressed in bone tissue.

## 2 Material and Methods

### 2.1 Peptide synthesis and modification

Exendin-4 (Ex-4) and exendin-4-Cys (Ex-4-Cys), bearing a linker and an additional cysteine residue at the c-terminus, were synthesized by Genecust (Genecust, Boynes, France) to >95% purity. Peptide purity and molecular mass were determined by reverse-phase HPLC and mass spectrometry. To obtain exendin-4-AF647 (Ex-4-AF647), Alexa Fluor 647 maleimide (#A20347, Invitrogen, Eugene, OR) was conjugated to Ex-4-Cys according to the manufacturer’s protocol. Similarly, exendin-4-bovine serum albumin (Ex-4-BSA) was generated by conjugation of maleimide-activated BSA (#77115, ThermoScientific, Waltham, MA) with Ex-4-Cys. Exendin-4-BSA-AF647 (Ex-4-BSA-AF647) was obtained by conjugation of Ex-4-BSA with Alexa Fluor 647 NHS ester (#A20006, Invitrogen) according to the manufacturer’s protocol.

### 2.2 Animal models

All procedures were carried out in accordance with the European Union Directive 2010/63/EU for animal experiments and were approved by the institutional animal care and use committee of the University of Angers (CEEA-PdL N°01740.01 and 6154-201607211130415v1) and University Paris Cité (CEEA40, authorization 12952-2017022013322777v4). Animals were randomly allocated to experimental groups and investigators were blinded during allocation, animal handling, and endpoint measurements. Unless otherwise stated, animals were housed in conventional facility in social groups (n=5/cages) and had access to water and standard rodent diet (A04, Safe, Augy, France) *ad libitum*. Sample size was not determined using a formal power calculation. Instead, a group size of eight animals per group was chosen based on prior studies from our laboratory, where this number was sufficient to detect significant bone fragility in ovariectomized (OVX) animals treated with saline. No attrition or dropout of animals occurred during the study; all animals completed the experimental protocol as planned.

#### 2.2.1 Mouse model of brain entry

Twenty 16-week-old ovariectomized female BALB/c mice (BALB/cJRj, RRID:IMSR_RJ:BALB-CJRJ) were purchased from Janvier Laboratories (Saint-Berthevin, France). Saline (n=5), Alexa-Fluor 647 (n=5, 700 pmoles), Ex-4-AF647 (n=5, 700 pmoles) or Ex-4-BSA-AF647 (n=5, 700 pmoles) were administered subcutaneously. Thirty minutes later, the animals were sacrificed by cervical dislocation and the brains carefully dissected and frozen in liquid nitrogen. Frozen brains were powdered using a tissue biopulverizer, and proteins were extracted by incubating brain powder with RIPA buffer at 4°C for 1 hour. Samples were centrifuged at 13,000 rpm for 30 minutes at 4°C, the supernatant collected, and protein concentration determined using a BCA protein assay kit (Pierce Biotechnology, Rockford, IL). Fluorescence was measured using an excitation wavelength of 645 nm and an emission wavelength of 670 nm on a SpectraMax M2 microplate spectrofluorometer (Molecular devices, Saint-Grégoire, France).

#### 2.2.2 Mouse model of ovariectomy-induced bone fragility

Ninety-six BALB/c female mice (BALB/cJRj) were purchased from Janvier Labs (Saint-Berthevin, France). We selected the BALB/c strain instead of C57BL/6 because BALB/c mice have a higher baseline trabecular bone mass and display a more pronounced reduction in trabecular bone content following ovariectomy (26). The degree of trabecular bone loss observed in the present study aligns with previously reported findings (27, 28), confirming that our results fall within the expected range. At 12 weeks of age, mice underwent either a sham operation (n=40) or bilateral (n=56) ovariectomy under general anesthesia, as previously described (27). Treatments began at 16 weeks of age and continued for 4 weeks. OVX mice were randomly allocated to receive vehicle, Ex-4, or Ex-4-BSA, administered either subcutaneously (sc) or intracerebroventricularly (icv). Mice in the SHAM group received only vehicle and served as non-osteopenic controls. A schematic overview of the experimental design is provided in the Supplementary Figure 1. For subcutaneous administration, an osmotic minipump (model 2004; Alzet, Rabalot, France) was inserted into a subcutaneous pouch on the dorsal surface of the animal under isoflurane anesthesia, and the animals received 0.05 mg/kg buprenorphine for the first 24 hours after surgery. For icv administration, animals were placed on a stereotaxic frame and a cannula was implanted in the lateral ventricle (X=-1.1mm, Y=-0.5mm and Z=-3mm positions from Bregma) under isoflurane anesthesia and received i.p. administration of xylazine 10 μg/kg. A catheter tube was connected from the cerebral perfusion cannula to an osmotic minipump (model 2004; Alzet). The minipump was inserted into a subcutaneous pocket on the animal’s dorsal surface. Eight animals were randomly assigned to each group. OVX mice were chronically perfused for 28 days with vehicle, ~700 pmol/day (sc) or 70 pmol/day (icv) Ex-4. These doses were chosen based on previous publications (15, 29, 30). Based on the activity of Ex-4-BSA at the GLP-1r, we employed a dose of 7 nmol/day (sc).

All animals received intraperitoneal administration of calcein green (10 mg/kg) 10 days and 2 days before sacrifice. Animals were housed in social groups and maintained in a 12 h:12 h light/dark cycle. They had free access to water and food. At necropsy, blood was collected by intracardiac aspiration into EDTA-treated tubes. Blood samples were centrifuged at 13,000 g for 15 min, aliquoted and stored at -80°C until plasma levels of exendin-4 (#EK-070-94, Phoenix pharmaceuticals, Burlingame, CA) and calcitonin (NBP3-06734, Bio-Techne Ltd, Abingdon, UK) were measured according to the manufacturer’s protocol. Plasma levels of the bone remodeling markers C-terminal telopeptide of collagen type I (CTx-I – RatLaps, Immunodiagnostic Systems Ltd, Boldon, UK) and N-terminal propeptide of type I collagen (P1NP - Rat/mouse P1NP, Immunodiagnostic Systems Ltd) were measured according to the manufacturers’ recommendation. Right femurs were wrapped in saline-soaked gauze and frozen at -20°C until use. Right tibiae were fixed in ethanol-based fixative and stored at 4°C until use. No specific inclusion or exclusion criteria were applied for animal selection. However, for ovariectomized animals, the efficacy of the surgery was verified by harvesting and weighing the uteri, with all animals exhibiting uterine atrophy consistent with successful ovariectomy. Additionally, correct implantation of the cerebral perfusion cannulas for icv-administered animals was verified at necropsy. All animals meeting these criteria were included in the study.

### 2.3 High resolution X-ray microCT

MicroCT analyses were performed at the proximal metaphysis of the right tibia and at the mid-diaphysis of the right femur with a Bruker 1272 microtomograph operated at 70 kV, 140 μA, 1000 ms integration time and imaging in ethanol 70. The isotropic pixel size was fixed at 4 μm, the rotation step at 0.25° and exposure was performed with a 0.5 mm aluminum filter. Hydroxyapatite phantoms (250 mg/cm^3^ and 750 mg/cm^3^) were used for calibration. Reconstruction of 2D projections was done with the NRecon software (Version 1.6.10.2). For tibial analysis, a trabecular volume of interest was located 0.5 mm below the growth plate at the proximal end and extended 2 mm down. Trabecular bone was separated from cortical bone with an automatic contouring script in CTan software (Version 1.20.8.0). The right femur was used for cortical microarchitecture. The region analyzed (0.5 mm) was centered at the midpoint between the third trochanter and the distal growth plate. Bone was segmented from soft tissue using global thresholding set at 300 mg/cm^3^ for trabecular bone and 700 mg/cm^3^ for cortical bone. All histomorphometrical parameters were measured with the CTan software according to guidelines and nomenclature proposed by the American Society for Bone and Mineral Research (31).

### 2.4 Histomorphometrical analysis

After microCT, right tibias were embedded undecalcified in polymethylmethacrylate (pMMA) at 4°C as previously reported (32). Seven micron-thick sections were made with a sledge microtome (Polycut S, Leica, Nanterre, France) prior to histoenzymatic detection of TRAP or Goldner trichrome staining. Dynamic histomorphometry was perfomed after counterstaining the bone matrix with calcein blue. All histomorphometrical parameters were measured with an in-house written application developed in Matlab R2023b (The Mathworks, Natick, CA, RRID:SCR_001622) according to guidelines and nomenclature proposed by the American Society for Bone and Mineral Research (33).

### 2.5 Assessment of bone strength

The whole-bone strength of right femurs was assessed by 3-point bending in accordance with published guidelines (34). Three-point bending strength was measured with a constant span length of 10 mm. Bones were tested in the antero-posterior axis with the posterior surface facing upward, centered on the support and the pressing force was applied vertically to the midshaft of the bone. Each bone was tested fully hydrated and at room temperature with a loading speed of 2 mm.min^-1^ until failure with a 500 N load cell on an Instron 5942 device (Instron, Elancourt, France) and the load-displacement curve was recorded at a 100 Hz rate by the Bluehill 3 software (Instron). Ultimate load, ultimate displacement, stiffness and work to fracture were calculated as indicated in (34). The yield point was determined as the point at which a regression line that represents a 10% loss in stiffness crosses the load-displacement curve. Post-yield displacement was computed as the displacement between yielding and fracture. Peak bending moment was calculated as one-half the ultimate load multiplied by one-half the span length (34). The peak bending moment is related to bone tissue material properties and bone midshaft geometry by the following equation:

$$M=\sigma_{b}\times \frac{I_{ML}}{c_{AP}}$$

Where M is peak moment bending, σ_b_ is bone tissue material strength (TMS), I_ML_ is the moment of inertia in the medio-lateral axis, and C_AP_ is the distance from the neutral axis to bone surface in the antero-posterior direction. Differences in peak moment bending that are not explained by I_ML_/C_AP_ are caused by alterations in tissue material properties.

### 2.6 Bone ECM material evaluation

Distal halves of the right femur were embedded undecalcified in pMMA after dehydration and infiltration. One micrometer-thick cross-section of the midshaft femur was cut with an ultramicrotome (Leica EM UC7, Leica microsystems, Nanterre, France) and deposited on BaF2 windows. Spectral analysis was performed, using a Bruker Hyperion 3000 infrared microscope coupled to a Bruker Vertex 70 spectrometer equipped with a single element mercury cadmium telluride (MCT) detector, between double calcein labels, as indicator of bone formation site. Mid-infrared spectra were recorded at a resolution of 4 cm^-1^ (Spectral range 850–2000 cm^-1^), with 32 accumulations in transmission mode. Background spectra were collected under identical conditions from the same BaF_2_ windows at the beginning and end of each experiment to ensure instrument stability. Post-processing was performed using Matlab R2023b (The Mathworks, Natick, CA) and included Mie scattering correction, pMMA subtraction, normalization and denoising (Savitzky-Golay algorithm, degree 2, span 9) prior to second derivative spectroscopy and curve fitting routines as previously reported in detail (35). A signal-to-noise ratio was computed in the region 1850-2000 cm^-1^ to ensure proper denoising and quality of FTIR spectra for subsequent second derivative and curve fitting. Bone ECM material parameters were: Phosphate/Amide ratio (area ratio of v1,v3 phosphate and amide I); mineral crystallinity/maturity (XST, area ratio of subbands located at subbands ~1030 cm^-1^ and ~1020 cm^-1^), carbonate/phosphate ratio (area ratio of v2 carbonate and v1,v3 phosphate), and collagen maturity (intensity ratio of subbands located at 1660 cm^-1^/1690 cm^-1^) (35).

### 2.7 Expression of Glp1r

Femurs from 12-week-old female BALB/c (BALB/cJRj) mice, purchased from Janvier Labs, were rapidly dissected, cleared of soft tissue before the distal ends were cut. Bones were centrifuged for 20 seconds at 16,000 g at 4°C, to remove bone marrow. Bone tissue was frozen in RNA later in liquid nitrogen and stored at -80°C until use. Hypothalamus, pancreas and sinus node region were used as positive controls and froze in RNA later. Liver and skeletal muscle were used as negative controls and processed as described above. After thawing, RNA was extracted by grinding tissue in Nucleozol (Macherey-Nagel, Hoerdt, France) and purifying total RNA with Nucleospin RNA columns (Macherey-Nagel) according to the manufacturer’s recommendations. Total RNA was reverse transcribed using the maxima first strand cDNA synthesis kit (Thermofisher scientific, Carlsbad, USA). Real-time quantitative polymerase chain reaction (qPCR) was performed using a Bio-Rad CFX 96 system and TaqMan gene expression assays (Thermofisher Scientific). Taqman assay ID # Mm00445292_m1 was used to assess the relative expression of mouse *Glp1r* versus mouse *B2m* (Taqman assay ID # Mm00437762_m1).

In addition, femurs from 5-week-old female BALB/c mice (BALB/cJRj) were rapidly dissected and fixed in formalin for 16 h before being decalcified in autoclaved 10% EDTA and embedded in paraffin following standard protocols. Tissues were cut into 5 μm sections and mounted on Superfrost plus gold glass slides (Thermofisher Scientific). In situ hybridization (ISH) was performed using the RNAscope v2 multiplex fluorescent reagent kit (Advanced Cell Diagnostics Inc., Newark, CA) and specific murine *Glp1r* (Assay No.: Mm-Glp1r #418851), murine Col1a1 (Assay No.: Mm-Col1a1 #319371) hybridization probes according to the manufacturer’s instructions and counterstained with DAPI. Slides were imaged with a Leica SP8 confocal microscope (Leica microsystems). Murine pancreases served as positive controls and were simultaneously processed as described above for bone samples and analyzed by RNAscope.

In addition, femurs and pancreases were isolated from three 13-week-old GLP-1R-Cre-eYFP mice, expressing cytosolic fluorescent eYFP only in cells expressing GLP-1r (36). Briefly, femurs and pancreases were fixed in 2.5% formaldehyde in 1X PBS, decalcified in 10% EDTA and embedded in paraffin. Five μm-thick sections were cut and mounted on Superfrost plus Gold glass slides (Thermofisher scientific). eYFP expression was assessed by immunohistochemistry (IHC) using anti-YFP (1/200 dilution, Abcam # Ab5450, RRID: AB_304897) incubated overnight at 4°C followed by an AlexaFluor 647-coupled secondary antibody (Thermofisher Scientific, # A66794). Slides were counterstained with DAPI and imaged with a Leica SP8 confocal microscope (Leica microsystems).

Publicly available RNA-sequencing datasets were also analyzed to investigate the expression of the murine *Glp1r* gene. We retrieved raw and/or processed data from the NCBI Gene Expression Omnibus (GEO) and the Sequence Read Archive (SRA) for the following datasets: GSE108892, PRJNA507938, and GSE154748. Reads were aligned to the mouse reference genome (GRCm38 or GRCm39, depending on dataset metadata) using STAR aligner (v2.7.9a). Gene-level expression quantification was carried out with featureCounts (v2.0.1). Normalized expression levels of *Glp1r* were extracted and data were inspected and visualized using R (v4.2.0) with the DESeq2 packages. Presence of transcripts was determined based on detectable expression based on gene threshold of TPM>0.1. For the PRJNA507938 dataset, only *Acp5*-positive samples were used for the detection of Glp1r. Expression of Glp1r was compared to that of Pth1r in osteoblast, osteocyte and osteoclast datasets.

### 2.8 Receptor binding and cAMP assays

The sequence encoding murine *Glp1r* was cloned into the pcDNA3.1(+) vector between the NheI and EcoRI sites of multiple cloning sites. CHO-K1 cells (RRID:CVCL_0214, ref CCL-61, ATCC, Molsheim, France) were grown and expanded at a 1:5 ratio in propagation medium containing F-12 supplemented with 10% FBS, 100 U/ml penicillin and 100 mg/ml streptomycin in a humidified atmosphere enriched with 5% CO2 at 37°C. CHO-K1 cells were transfected with the plasmid encoding the murine GLP-1r (mGLP-1r) using Invitrogen™ Lipofectamine™ 3000 as recommended by the manufacturer. For binding assay, 24h post-transfection, cells were detached and plated at a density of 6x10^4^ cells/cm^2^ in black 96 well plates with clear bottom (Ibidi GmbH, Martinsried, Germany). After 24h, cells were exposed to various concentrations of Ex-4 or Ex-4-BSA in the presence of 10^-6^M Fam-[D-Ala2]-GLP-1 in α-MEM supplemented with 0.1% bovine serum albumin. Equilibrium binding was achieved overnight at 37°C. Cells were then washed twice with PBS and solubilized in 0.1M NaOH. Fluorescence was read with on a SpectraMax M2 microplate spectrofluorometer with excitation and emission wavelength set up at 490 nm and 525 nm respectively.

For activation assay, plasmid encoding the mGLP-1r and the Epac-S-H74 probe, a validated cAMP FRET biosensor (37), were transfected with Invitrogen™ lipofectamine™ 3000 as described above. Forty-eight hours later, transfected cells were incubated in HEPES buffered saline in the presence of Ex-4-based peptides for 30 minutes. Donor excitation was made at 460 nm, donor emission was collected at 480 nm and acceptor emission at 560 nm with a SpectraMax M2 microplate spectrofluorometer. FRET was expressed as the ratio between donor and acceptor signals. The FRET ratio was standardized at 1 with vehicle. An increase in FRET ratio suggests an augmentation in intracellular cAMP levels and hence receptor activation.

### 2.9 Bone marrow-derived mesenchymal stem cells differentiation

Femurs and tibias were isolated from 12-week-old female BALB/c mice (BALB/cJRj; Janvier Labs, Saint-Berthevin, France). Bones were rapidly dissected and cleared of soft tissue. The distal epiphyses were removed, and the marrow cavities were flushed with sterile PBS to collect bone marrow cells. Cells were suspended in growth medium composed of α-MEM supplemented with 10% fetal bovine serum, 100 U/mL penicillin-streptomycin, and 0.25 μL/mL Fungizone (Thermo Fisher Scientific), and seeded into 6-well plates at a density of 2.5 × 10^6^ cells/well. Cultures were maintained at 37°C in a humidified incubator with 5% CO_2_. Cells remained undisturbed for 5 days to promote adherence. On day 5, growth medium was replaced with osteogenic differentiation medium consisting of the same basal medium supplemented with 50 μg/mL ascorbic acid, 2.5 mM β-glycerophosphate, and vehicle, 1 nM exendin-4 or 50 nM PTH. Media were refreshed on day 7 and then every 2 days thereafter.

On day 14, cells were washed with PBS and subjected to three freeze-thaw cycles. ALP was extracted by scraping the cells into 250 μL of 0.2% (v/v) Nonidet P-40 (Sigma-Aldrich). Enzymatic activity was measured using the fluorescent substrate 4-methylumbelliferyl phosphate (Sigma-Aldrich) as previously described (38).

On day 21, cells were fixed with 3.7% (w/v) formaldehyde in PBS for 10 min, rinsed with PBS, and stained with 1.5% (w/v) Alizarin Red S for 5 min. Plates were rinsed three times with PBS. Bound dye was extracted using 10% acetic acid, neutralized with 10% ammonium hydroxide, and absorbance was measured at 550 nm using a spectrophotometer.

### 2.10 Osteoclast Differentiation Assay

To investigate the potential effect of Exendin-4 (Ex-4) on osteoclast differentiation, we used splenocytes isolated from BALB/c ovariectomized (OVX) mice (BALB/cJRj; Janvier Labs, Saint-Berthevin, France). Mice were ovariectomized at 12 weeks of age, and splenocytes were isolated 4 weeks post-ovariectomy. The splenocytes were cultured in osteoclast differentiation medium (α-MEM supplemented with 10% FBS, 100 U/mL penicillin/streptomycin, 50 ng/mL macrophage colony-stimulating factor (M-CSF, Bio-Techne, Noyal-Chatillon-sur-Seiche, France), and 30 ng/mL receptor activator of NF-κB ligand (RANKL, Bio-Techne) in the presence or absence of 10 nM Exendin-4. D-Ala2-GIP at a concentration of 10 nM was used as positive controls, as previously reported (39). Cells were cultured for 7 days, with media changes every 2 days. Osteoclast differentiation was evaluated by TRAP staining, as previously described (39).

### 2.11 Statistical analysis

Statistical analyses were performed using GraphPad Prism 8.0 (GraphPad Software, La Jolla, CA, USA, RRID:SCR_002798). For each investigated parameter (e.g., BV/TV), an ordinary one-way ANOVA was conducted to assess the effect of the experimental group. The single factor was the experimental group, a between-subjects factor with three levels: Sham + vehicle, OVX + vehicle, and OVX + Ex-4. Since only one factor was analyzed, no interaction term was applicable. Tukey’s multiple comparisons post hoc test was used to assess pairwise differences between group means following each ANOVA. Data normality was assessed using the Shapiro-Wilk test, and homogeneity of variances was verified using the Brown-Forsythe test. Outliers were identified using the ROUT method (Q = 1%). Only exact p-values are reported for each ANOVA and post hoc comparison. Data are presented as mean ± standard deviation (SD). Statistical significance was set at p < 0.05. A linear regression analysis was performed to assess the relationship between collagen maturity and tissue material strength or energy-to-fracture. The strength of the association was quantified using Pearson’s correlation coefficient (r), and the exact p-value is reported. No additional regression parameters (e.g., slope, intercept, or R^2^) were included. Binding at the mGLP-1r was analyzed using a non-linear regression with the one-site Fit log IC50 model.

## 3 Results

### 3.1 Subcutaneous administration of exendin-4 improves bone strength and bone material properties in OVX animal

**Figure 1:**
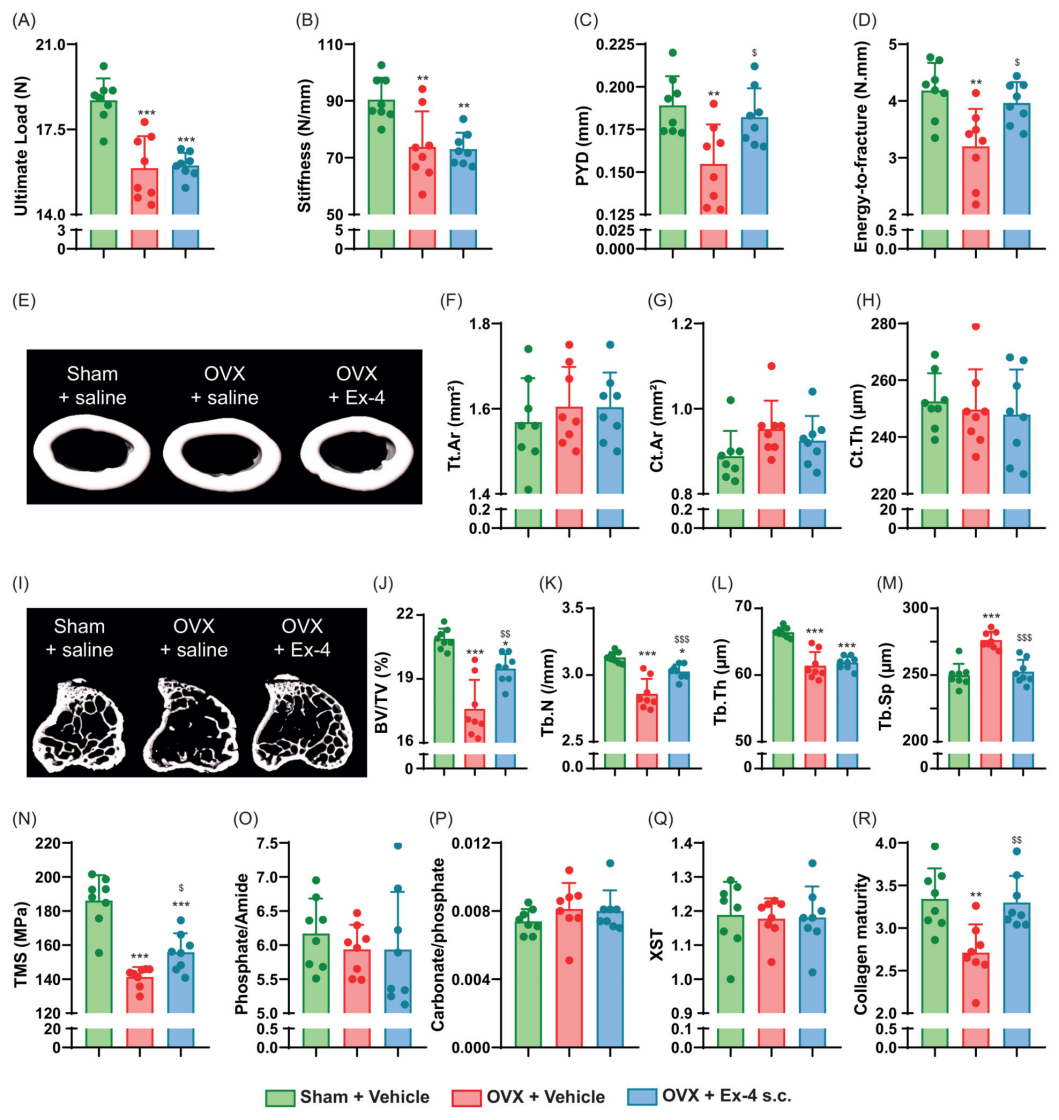
Investigation of bone phenotype following subcutaneous administration of exendin-4. (A-D) Mechanical properties of the femur and (E-H) cortical bone microstructure were investigated at the midshaft femur by 3-point bending and microCT. (I-M) Trabecular microstructure was investigated at the proximal tibia metaphysis. (N-R) Bone material strength and properties were investigated at the midshaft femur. In panels A-R, Green bars = Sham + vehicle, red bars = OVX + vehicle and blue bars = OVX + Ex-4. Statistical analyses were performed with one way ANOVA with Tukey’s multiple comparisons test. *: p<0.05; **: p<0.01; ***: p<0.001 vs. Sham+Vehicle. ^$^: p<0.05; ^$$^: p<0.01; ^$$$^: p<0.001 vs. OVX+Vehicle. N=8 animals/group.

Ex-4 was administered peripherally via subcutaneous implantation of osmotic minipumps. No significant changes in body mass were encountered between vehicle- or s.c. Ex-4-treated OVX animals (Supplementary Figure 2). As expected, ovariectomy resulted in a significant deterioration in the mechanical response of long bones, represented by decreases in ultimate load (-15%, p<0.001), stiffness (-18%, p=0.004), post-yield displacement (-18%, p=0.005) and energy-to-fracture (-24%, p=0.003) (Figures 1A-D). Subcutaneous administration of Ex-4 improved plastic deformation of the femurs, as shown by significant increases in post-yield displacement (18%, p=0.025) and energy-to-fracture (24%, p=0.020). On the other hand, subcutaneous administration of Ex-4 did not prevent the impact of ovariectomy on ultimate load (p=0.817) and stiffness (p=0.984).

To better understand whether the positive effects of Ex-4 were related to changes in femur microstructure, we assessed bone microarchitecture at the mid-diaphysis level (Figures 1E-H). Interestingly, OVX animals showed no significant changes in cortical bone microstructure compared with sham animals. This finding was previously reported by others and ourselves (27, 28). Similarly, subcutaneous administration of Ex-4 did not alter cortical bone microstructure in OVX animals despite positive effects on biomechanics. Trabecular microarchitecture was assessed at the proximal metaphysis of the tibia rather than at the distal femur metaphysis, as previous studies reported bigger change at this specific site in OVX animals (26, 28) (Figure 1I-1M). As expected, OVX led to decreases in bone mass represented by lower BV/TV (-16%, p<0.001), Tb.N (-9%, p<0.001) and Tb.Th (-8%, p<0.001). Interestingly, subcutaneous administration of Ex-4 resulted in higher BV/TV (11%, p=0.002) and Tb.N (6%, p<0.001). Histological evaluation revealed no effects of ovariectomy on dynamic morphometry or osteoid parameters (Table 1). On the other hand, the number of osteoclasts and osteoclast surfaces were significantly increased in OVX+vehicle animals (76%, p<0.001) and restored after subcutaneous administration of Ex-4 (-41%, p<0.001). Plasma circulating levels of calcitonin were also significantly reduced in vehicle-treated ovariectomized animals (-31%, p=0.001) and significantly increased in OVX animals treated with subcutaneous Ex-4 (152%, p<0.001) (Table 1). Plasma levels of CTX-I were significantly elevated in OVX+vehicle animals as expected (+78%, p<0.001) and reduced after subcutaneous administration of Ex-4 (-20%, p=0.012).

We then calculated tissue material strength to determine whether the mechanical response was related to changes in bone material. Interestingly, OVX animals showed a significant reduction in tissue material strength compared to sham animals (-24%, p<0.001) which was partially reversed by Ex-4 administration (11%, p=0.043), suggesting that the main effect of Ex-4 on cortical bone strength was related to changes in bone material (Figure 1N). At the material level, the bone mineral was not affected by OVX or subcutaneous administration of Ex-4 (Figures 1O-Q). In contrast, collagen maturity, representing the ratio of pyridinoline to dihydroxylysinonorleucine, was significantly lower in vehicle-treated ovariectomized animals (-19%, p=0.003) and significantly improved by subcutaneous administration of Ex-4 (18%, p=0.005) (Figure 1R). Interestingly, collagen maturity correlated linearly with tissue material strength (r = 0.50, p=0.012) and energy-to-fracture (r =0.63, p =0.001).

### 3.2 GLP-1r receptor is not expressed in bone

**Figure 2:**
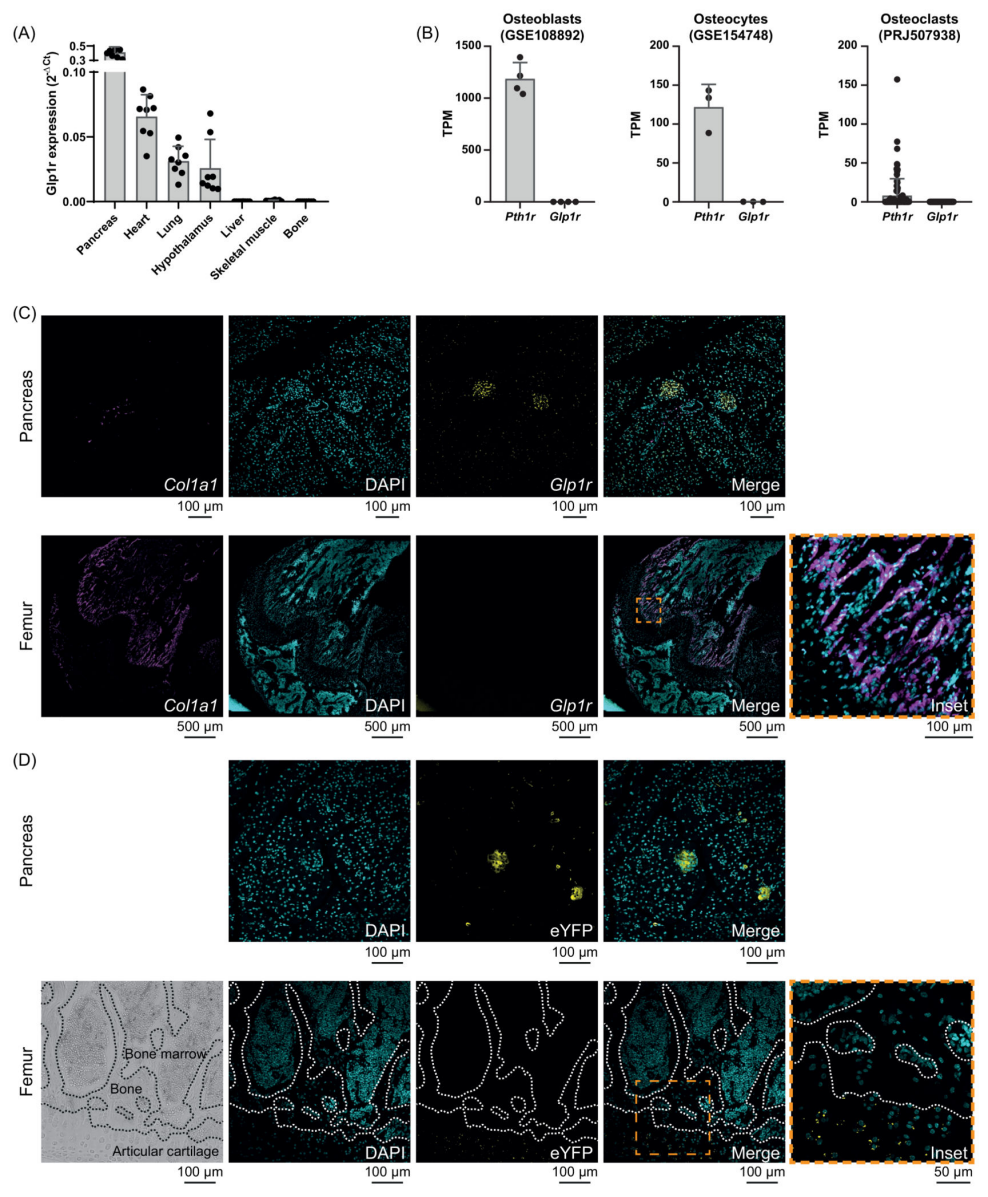
Expression of *Glp1r* in bone. *Glp1r* expression was investigated by (A) qPCR (n=8 animals/group). Pancreases, hearts, lung and hypothalamus were used as positive controls. Liver and skeletal muscle were used as negative controls. (B) Presence of *Glp1r* transcripts in publicly available RNA-seq dataset was investigated and compared with transcripts of *Pth1r* in osteoblast, osteocyte and osteoclast datasets. (C) Glp1r expression was investigated by in situ hybridization using the RNAscope technology (n=5 animals/group). The orange dashed box indicates the area shown at higher magnification in the inset. (D) Expression of *Glp1r* was investigated at the gene level in pancreas and bone extracted from GLP-1R-Cre-eYFP mice. The presence of eYFP-positive cells indicates that these cells had previously expressed the *Glp1r* gene (n=3 animals/group). Dashed lines represent the contour of bone trabecula. The orange dashed box indicates the area shown at higher magnification in the inset. All panels are qualitative and intended to illustrate tissue-specific expression patterns ; no statistical analysis was applied.

As exendin-4 exerts positive effects on the properties of bone material and in particular on collagen maturity, we wanted to determine whether Ex-4 acted directly on osteoblasts. As expected, *Glp1r* expression was found in pancreas, heart, lung and hypothalamus (positive controls) and absent in liver and skeletal muscle (negative controls). Interestingly, we were unable to detect *Glp1r* expression in bone by qPCR (Figure 2A). We next sought to ascertain whether Glp1r transcripts could be retrieved from publicly available RNA-seq datasets. Once again, we were unable to unequivocally demonstrate the presence of Glp1r transcripts (Figure 2B). We also explored *Glp1r* expression by in situ hybridization with the RNAscope assay. Although *Glp1r* expression was found in pancreatic islets, we failed to demonstrate the presence of *Glp1r* expression in bone marrow, in *Col1a1*-positive osteoblasts, osteocytes or osteoclasts (Figure 2C). We also investigated the presence of eYFP-positive cells in the pancreas and long bones of GLP-1R-CRE-eYFP mice (Figure 2D). Again, we demonstrated the presence of eYFP-positive cells in pancreatic islets but failed to demonstrate the presence of eYFP-positive cells in bone marrow or bone tissue. However, several articular chondrocytes were eYFP-positive, suggesting that during articular chondrocyte differentiation, the *Glp1r* promoter was activated and led to transcriptional and translational expression of eYFP. Overall, these data suggest that the positive effects of subcutaneous Ex-4 administration on bone mechanics and fracture resistance are indirect. Furthermore, treatment of mouse bone marrow-derived mesenchymal stem cell (BMMSC) cultures with Exendin-4 (Ex-4) did not result in any significant increase in alkaline phosphatase (ALP) activity or extracellular matrix mineralization (Supplementary Figure 3). Moreover, splenocytes were cultured with osteoclast differentiation factors in the presence or absence of Exendin-4 (Ex-4). No significant differences in osteoclast differentiation were observed (Supplementary Figure 4), indicating that Exendin-4 does not impact osteoclast differentiation under these experimental conditions.

### 3.3 Intracerebroventricular administration of exendin-4 improves bone strength and bone material properties in OVX animal

**Figure 3:**
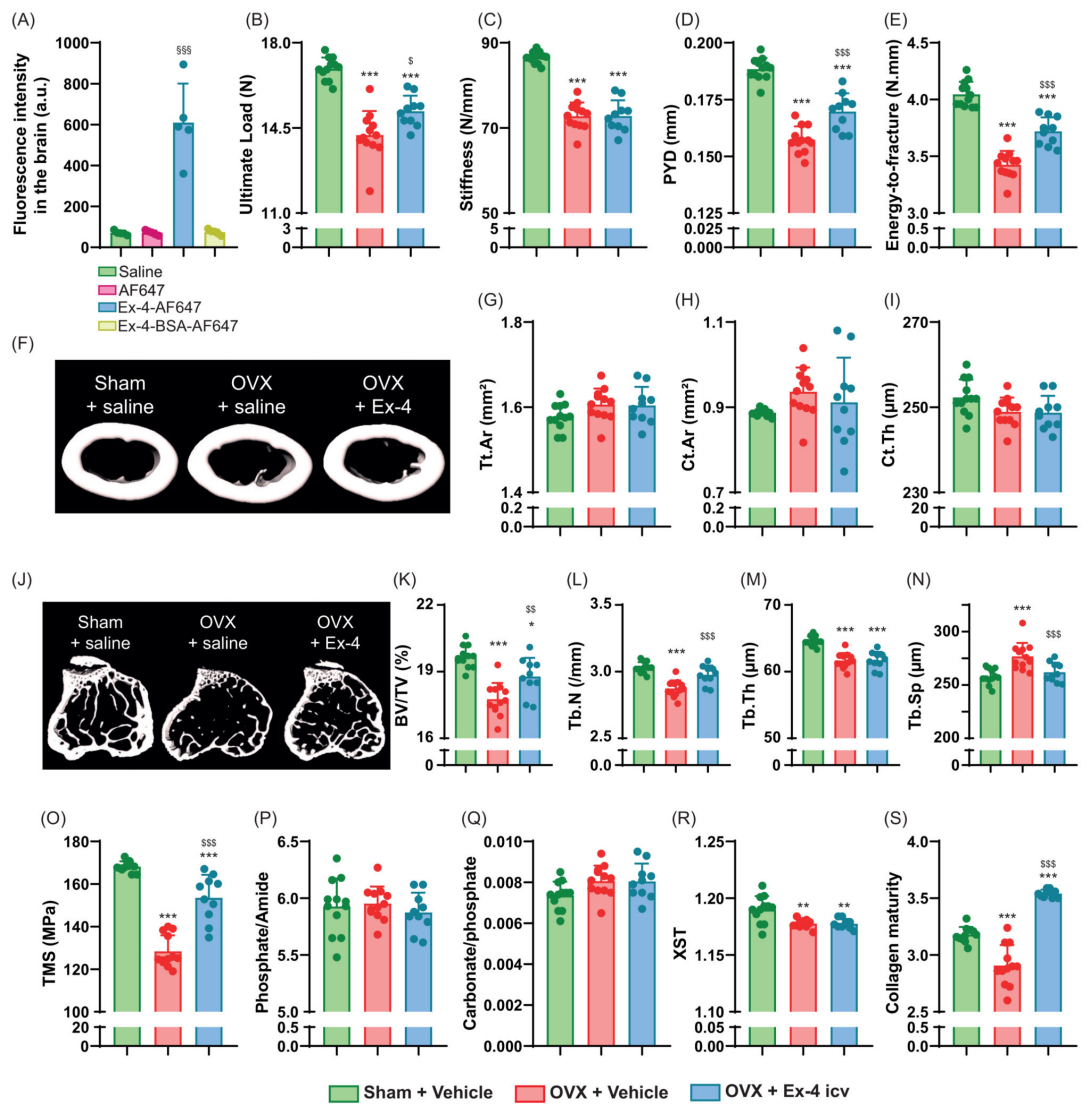
Investigation of bone phenotype following intracerebroventricular administration of exendin-4. **(A)** Crossing of the blood-brain barrier was investigated by using fluorescently labelled Ex-4. **(B-E)** Mechanical properties and (F-I) cortical bone microstructure were investigated at the midshaft femur by 3-point bending and microCT. (J-N) Trabecular microstructure was investigated at the proximal tibia metaphysis by microCT. (O-S) Bone material strength and properties were investigated at the midshaft femur. In panels B-S, Green bars = Sham + vehicle, red bars = OVX + vehicle and blue bars = OVX + Ex-4. Statistical analyses were performed with one way ANOVA with Tukey’s multiple comparisons test. ^§§§^: p<0.001 vs. saline; *: p<0.05; **: p<0.01; ***: p<0.001 vs. Sham+Vehicle. ^$^: p<0.05; ^$$^: p<0.01; ^$$$^: p<0.001 vs. OVX+Vehicle. N=8 animals/group.

As liraglutide, another GLP-1r analogue was able to enhance bone resistance to fracture in a model of streptozotocin-induced type 1 diabetes mellitus (16) characterized by chemical destruction of pancreatic islets, we hypothesized that Ex-4 mediates its effects through activation of receptors in other tissues, including the CNS. We first examined whether Ex-4 could enter the brain by using Ex-4-AF647. Within 30 minutes of subcutaneous administration, Ex-4-AF647 was detected in brain extracts, suggesting that not only Ex-4-AF647, but also possibly Ex-4, were able to access the CNS at least in circumventricular organs and adjacent brain nuclei (Figure 3A).

We then administered saline or Ex-4 intracerebroventricularly (icv) for 4 weeks via a cannula inserted into the lateral ventricle and connected it to a peripheral osmotic minipump in ovariectomized animals. Ovariectomized mice treated with icv saline exhibited a significant reduction in ultimate load (-19%, p<0.001), stiffness (-16%, p<0.001), post-yield displacement (-16%, p<0.001) and energy-to-fracture (-15%, p<0.001) compared to sham control animals (Figures 3B-E). Interestingly, icv administration of Ex-4 resulted in a significant improvement in ultimate load (7%, p=0.010) and plastic deformation, represented by higher post-yield displacement (8%, p<0.001) and energy-to-fracture (8%, p<0.001). We then tested whether the positive effects on biomechanics following icv administration of Ex-4 were due to changes in cortical bone microstructure. However, here again and similarly to what was found for subcutaneous administration of Ex-4, we were unable to demonstrate significant changes in cortical bone microstructure (Figures 3F-I). The microstructure of the trabecular bone was studied in the proximal metaphysis of the tibia (Figs. 3J-N). Interestingly, vehicle-treated OVX animals showed a decrease in BV/TV (-10%, p<0.001), Tb.N (-5%, p<0.001) and Tb.Th (-5%, p<0.001), confirming the results observed in the above-mentioned subcutaneous study. Intracerebroventricular administration of Ex-4 reversed alterations in BV/TV (+6%, p=0.005) and Tb.N (4%, p<0.001). Histological analyses again revealed that the main effects of OVX concerned osteoclast numbers (73%, p=0.003) and osteoclast surfaces (107%, p<0.001) (Table 2). Intracerebroventricular administration of Ex-4 reduced the number of osteoclasts (-51%, p<0.001) and osteoclast surfaces (-45%, p<0.001), but had no effects on circulating calcitonin levels. Plasma levels of CTX-I were significantly elevated in OVX+vehicle animals (+85%, p<0.001) and reduced after icv administration of Ex-4 (-38%, p=0.005). We then calculated tissue material strength and found that ovariectomized animals treated with icv saline had significantly lower tissue material strength than sham animals (-24%, p<0.001) (Figure 3O). Interestingly, ovariectomized animals treated with icv Ex-4 showed a significant improvement in this parameter (9%, p=0.005), suggesting an effect of Ex-4 on bone material properties via a central relay. At material level, vehicle-treated OVX animals showed normal mineral properties (Figures 3P-R). In contrast, collagen maturity was significantly reduced in OVX animals (-8%, p<0.001) (Figure 3S). Intracerebroventricular administration of Ex-4 in OVX animals improved collagen maturity (22%, p<0.001). Interestingly, collagen maturity correlated linearly with tissue strength (r=0.70, p<0.0001) and energy-to-fracture (r=0.39, p=0.021).

### 3.4 Subcutaneous administration of exendin-4-BSA failed to improve bone strength and bone material properties in OVX animal

**Figure 4:**
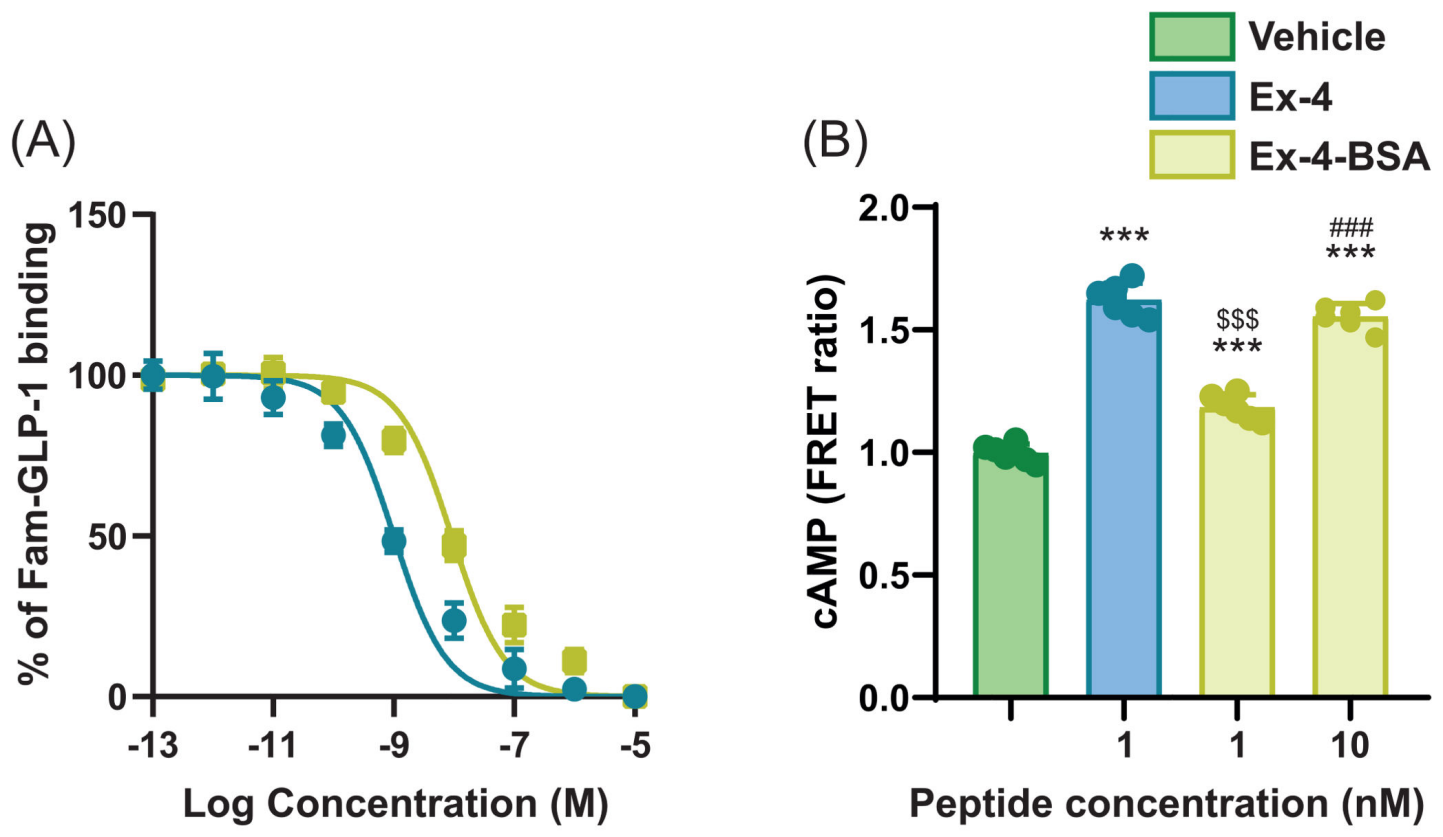
Binding to and activation of the mGLP-1r by Ex-4-BSA. (A) Competitive binding assay of Ex-4-BSA in mGLP-1r-transfected CHO-K1 cells (n=6 replicates/concentration/treatment). (B) Cyclic AMP production was detected by FRET using the H74 cAMP biosensor probe in mGLP-1r-transfected CHO-K1 cells (n=6 replicates/concentration/treatment). Production of cAMP is seen by increasing FRET ratio. Statistical analyses were performed with one way ANOVA with Tukey’s multiple comparisons test. ***: p<0.001 vs. Vehicle; ^$$$^: p<0.001 vs. Ex-4 and ^###^: p<0.001 vs. 1 nM Ex-4-BSA.

**Figure 5:**
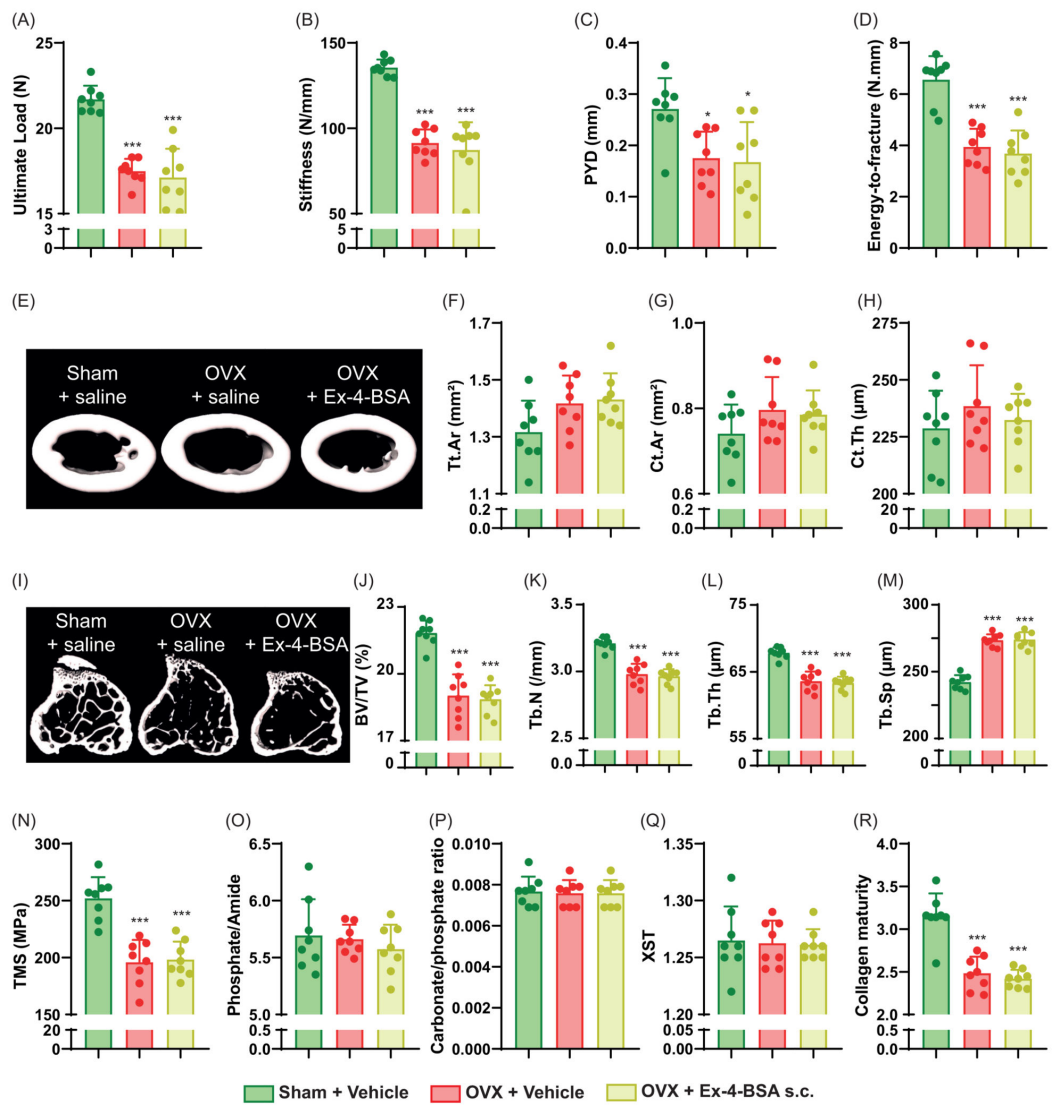
Investigation of bone phenotype following subcutaneous administration of exendin-4-BSA. Mechanical properties of the femur (A-D) and cortical microstructure (E-H) were investigated at the midshaft femur. (I-M) Trabecular microstructure was investigated at the proximal tibia metaphysis. (N-R) Bone material strength and properties were investigated at the midshaft femur. In panel A-Q, Green bars = Sham + vehicle, red bars = OVX + vehicle and yellow bars = OVX + Ex-4-BSA. Statistical analyses were performed with one way ANOVA with Tukey’s multiple comparisons test. *: p<0.05; **: p<0.01; ***: p<0.001 vs. Sham+Vehicle. N=8 animals/group.

To verify that Ex-4 mode of action on bone biomechanics and material properties involves activation of a central relay, we designed the Ex-4-BSA agonist, corresponding to Ex-4 coupled to bovine serum albumin. We first verified that Ex-4-BSA was unable to enter the brain and as expected, Ex-4-BSA-AF647 was not found in brain extracts (Figure 3A). Because of potential steric interference of the BSA moiety with GLP-1r interaction we then assessed whether Ex-4-BSA could still bind to murine GLP-1r (Figure 4A). Compared with Ex-4, Ex-4-BSA showed a ~1 log difference in binding activity (EC50 = 9.8 x 10^-9^ M ± 3.5 x 10^-9^ M vs. 1.1 x 10^-9^ M ± 0.4 x 10^-9^ M, p<0.001) (Figure 4A). We next ascertained whether Ex-4-BSA was also capable of activating the mGLP-1r (Figure 4B). Ex-4-BSA was administered in cultures of CHO-K1 cells transfected with the mGLP-1r and the H74 cAMP biosensor. As compared with Ex-4, Ex-4-BSA was capable of inducing the intracellular production of cAMP but with a concentration ~10 times higher, suggesting that it could still activate the mGLP-1r (Figure 4B). Ex-4-BSA was then subcutaneously administered for 4 weeks with an osmotic minipump into ovariectomized mice at a concentration of 7 nmol/day. Interestingly, Ex-4-BSA did not improve any of the biomechanical parameters, that were all affected by ovariectomy (Figures 5A-D). Similarly, Ex-4-BSA had no effect on cortical and trabecular bone microarchitectures (Figures 5E-M). Histological analyses failed to evidence any effects of Ex-4-BSA on the number of osteoclasts or osteoclast surfaces. However, Ex-4-BSA induced an increase in circulating calcitonin levels (152%, p<0.001) suggesting that Ex-4-BSA was active at peripheral sites (Table 3). Furthermore, plasma levels of CTX-I were significantly elevated in OVX+vehicle animals (+91%, p<0.001). However, subcutaneous administration of Ex-4-BSA did not reduced the circulating levels of CTX-I (p=0.997), suggesting that a central action is required to reduced bone resorption. Subcutaneous administration of Ex-4-BSA did not improve tissue material strength (p=0.962) (Figure 5N). Furthermore, at the material level, Ex-4-BSA did not modify collagen maturity in OVX animals (p=0.802) (Figure 5R).

## 4 Discussion

Bone fragility is on the rise worldwide, and new strategies are needed to better manage patients at risk of bone fracture. Among these, the repurposing of GLP-1r agonists, approved for the treatment of type 2 diabetes and obesity, could represent a valuable solution.

In the present study, we unambiguously demonstrated that subcutaneous or intracerebroventricular administration of the GLP-1r agonist, exendin-4, was capable of improving not only the microstructure of trabecular bone, but also the strength of cortical bone, through increased collagen maturity. We have also shown that Ex-4 enters the brain. These results were not observed in animals treated with Ex-4-BSA, suggesting that the action of Ex-4 is central. Consistent with a central relay, we failed to demonstrate GLP-1r expression in bone tissue. Furthermore, direct infusion of exendin-4 to osteoblast or osteoclast cultures in vitro failed to exhibit any effects supporting the lack of receptor expression in bone cells. By using the GLP-1R-Cre-eYFP mice, our group and others have previously reported the expression of the GLP-1r in the arterial walls of the kidneys and lungs, cardiac myocytes of the sinus node, gastric antrum and pylorus, enteric neurons, vagal and dorsal root ganglia, β-cells of the pancreas and c-cells of the thyroid gland (36, 40). We also previously and extensively reported the expression of the GLP-1r in central regions and have chosen not to repeat it again in the present manuscript. Nevertheless, centrally, GLP-1r is expressed in the olfactory bulb, amygdala, preoptic gland, amygdala, preoptic area, nucleus accumbens, hypothalamic arcuate nucleus, paraventricular nucleus, dorsomedial nucleus, lateral hypothalamic nucleus, supraoptic nucleus, postrema area, nucleus tractus solitarius and lateral reticular nucleus (36, 40–42). It should be noted that we observed a slight but significant expression of eYFP protein in articular chondrocytes from GLP-1R-Cre-eYFP mice, suggesting that the *Glp1r* promoter was activated during articular chondrocyte differentiation. However, the lack of *Glp1r* transcripts in ISH studies in younger animals suggests that *Glp1r* was not yet expressed at the time of sacrifice and may appear between 5 and 13 weeks of age. This observation highlights the importance of interpreting the eYFP signal as a marker of historical *Glp1r* promoter activity, which may not reflect current expression levels. Nevertheless, GLP-1r expression in articular cartilage has also been confirmed by others (43, 44). Pancreatic GLP-1r activation is unlikely to be responsible for the observed increase in collagen maturity and bone strength upon Ex-4 administration, as animals with significant pancreatic islet destruction by streptozotocin still showed the same type of response (16). Someone might criticize us for not investigating the presence of the GLP-1r at the protein level other than by using the GLP-1R-Cre-eYFP mice. However, it should be noted that no commercially available antibody specific to the murine GLP-1r exists, and several researchers, including ourselves, have previously reported this issue (24, 25). The absence of GLP-1r-specific antibodies prevents investigation by immunohistochemistry or Western blot; therefore, gene expression analysis (qPCR or ISH) remains the most reliable approach, in complement with the GLP-1R-Cre-eYFP mice, to explore the tissue expression of murine GLP-1r. This is very important has previous reports showing the presence of the GLP-1r in bone and bone cells used an unreliable antibody (abcam #ab39072) and as such, the unambiguous presence of the receptor is uncertain (20, 45). Nevertheless, and in agreement with the current study, several authors failed to identify the presence of *Glp1r* transcripts in osteoblast cells (24, 46).

Global *Glp1r* KO animals show alterations in bone strength, reduced trabecular bone mass and material properties, suggesting GLP-1 control of these bone functions (6, 7). These observations formed the basis for investigating the potential utility of a GLP-1r agonist for the management of bone fragility. Ex-4 has already been administered subcutaneously in preclinical models of bone fragility, including the OVX model used in the present study. Previous studies have reported positive effects of Ex-4 that increased bone mineral density, trabecular microstructure and circulating calcitonin levels and reduced osteoclast parameters, resulting in greater bone strength (15). Our study recapitulates these findings, but more importantly highlights that although Ex-4 can stimulate calcitonin secretion by the thyroid gland in rodents (47), the effects on trabecular and cortical bone microstructure, material properties and strength are due to activation of a central relay. Furthermore, despite a significant rise in circulating calcitonin levels in response to Ex-4-BSA, this GLP-1r agonist is unable to affect bone physiology, suggesting that the positive effects of Ex-4 on bone may be independent of calcitonin secretion in rodents. This mechanism had already been reported in Pereira et al. investigating the impact of GLP-1r agonists in OVX animals (20). Interestingly, intracerebroventricular administration of Ex-4 recapitulated the greater collagen maturity observed after subcutaneous administration, suggesting that central GLP-1r is required to modulate the quality of the bone material component. This hypothesis is reinforced by the lack of effect of Ex-4-BSA, which does not enter the brain, on bone material properties. This result is in line with a previous in vitro study which showed no direct positive effects of GLP-1 on collagen maturity (24). Bone fragility is governed by the triad of bone mass - bone microstructure - bone material properties. The idea that bone mass can be controlled by central relays is not new, and dates back to the early 2000s with the pioneering work of the Karsenty lab (48) which has now been replicated and extended by several investigators worldwide and has been the subject of several reviews over the last decade (49, 50). However, to our knowledge, the present study is the first report of bone quality control by a central relay. Of course, further work is needed to decipher which central region is involved in this process and how centrally generated signals can be projected to the skeleton.

Furthermore, although the GLP-1 sequence is conserved between rodents and humans, the tissue distribution of GLP-1r differs between the two species, with the example of *Glp1r* expression in thyroid C cells in rodents but not in non-human primates or humans (47). In the present study, Ex-4-BSA failed to exhibit significant improvement in collagen maturity and hence bone strength, despite increased secretion of calcitonin in animals and activity at the murine GLP-1r in transfected CHO cells. This suggests that the beneficial effects of Ex-4, and possibly GLP-1, on collagen maturity and bone strength is not mediated by either calcitonin secretion or peripheral action, but rather by activation of a central relay. With respect to the lack of expression of *GLP1R* in human thyroid C-cells, it would be interesting to investigate whether the central action of Ex-4 is preserved in humans and could represent a viable option to treat bone fragility due to poor bone material quality.

A limited number of studies have investigated whether endogenous GLP-1 or GLP-1r agonists were capable of entering the brain. ^125^I-labeled Ex-4 has been shown to rapidly enter the brain in a non-saturable manner at doses below 30 nmol/kg (51). Furthermore, CNS access of intravenously infused radiolabeled [Ser8]GLP-1, a stable analogue of GLP-1, is not inhibited by excess doses of unlabeled [Ser8]GLP-1 or by the GLP-1r antagonist exendin (9–39) in mice (52). Taken altogether, these studies might support a brain entry through adsorptive transcytosis across brain endothelial cells. The lack of active receptor-mediated transport of GLP-1 is further supported by recent transcriptomic data failing to undoubtedly show the expression of *Glp1r* in hypothalamic endothelial cells or cells of the neurovascular unit (53). Brain entry is not limited to Ex-4 and lipidation of GLP-1r agonists does not seem to be a problem for brain entry as lipidated Ex-4, liraglutide and semaglutide have also been reported to enter the brain parenchyma after peripheral administration (54–56).

In conclusion, the present study identified a new mode of action of exendin-4 through a central relay in order to induce significant improvement in bone strength through action at the bone material level and collagen maturity. Whether such a mechanism of action is preserved in humans remains to be elucidated.

## Supplementary Material

Supplementary Figures

## Figures and Tables

**Table 1:** Bone histomorphometry at the proximal tibia metaphysis and plasma levels of calcitonin after subcutaneous administration of Ex-4

	Sham + Vehicle (n=8)	OVX + Vehicle (n=8)	OVX + Ex-4 (n=8)
O.Th (μm)	6.1 ± 2.2	4.7 ± 1.9	4.8 ± 0.8
OS/BS (%)	2.4 ± 1.8	2.2 ± 0.5	2.4 ± 1.0
MAR (μm/day)	2.55 ± 0.57	2.19 ± 0.49	2.30 ± 0.35
MS/BS (%)	14.1 ± 2.4	13.5 ± 0.7	13.0 ± 1.3
BFR/BS (μm^3^/μm^2^/day)	0.360 ± 0.106	0.281 ± 0.068	0.301 ± 0.059
N.Oc/B.Pm	1.08 ± 0.25***	1.87 ± 0.38	1.11 ± 0.26***
Oc.S/BS (%)	1.04 ± 0.11***	1.90 ± 0.24	0.99 ± 0.28***
Calcitonin (pg/ml)	64 ± 4**, ^$$$^	44 ± 7	111 ± 15***
CTx-I (ng/ml)	8.2 ± 1.0***, ^$$^	14.6 ± 2.4	11.7 ± 2.0*
P1NP (ng/ml)	20.5 ± 3.8*	26.4 ± 3.7	25.2 ± 4.0

One way ANOVA with Tukey’s multiple comparisons test.

* p<0.05,

** p<0.01

*** p<0.001 vs. OVX+Vehicle;

$$ p<0.01

$$$ p<0.001 vs. OVX+Ex-4

**Table 2:** Bone histomorphometry at the proximal tibia metaphysis and plasma levels of calcitonin and exendin-4 after intracerebroventricular administration of Ex-4.

	Sham + Vehicle (n=8)	OVX + Vehicle (n=8)	OVX + Ex-4 (n=8)
O.Th (μm)	5.3 ± 2.1	5.7 ± 2.9	4.7 ± 0.7
OS/BS (%)	1.7 ± 1.8	2.7 ± 1.4	3.0 ± 2.2
MAR (μm/day)	2.36 ± 0.49	2.19 ± 0.40	2.17 ± 0.41
MS/BS (%)	12.1 ± 1.9	13.0 ± 1.0	12.9 ± 1.2
BFR/BS (μm^3^/μm^2^/day)	0.278 ± 0.068	0.285 ± 0.062	0.282 ± 0.065
N.Oc/B.Pm (mm^-1^)	1.20 ± 0.63**	2.07 ± 0.63	1.01 ± 0.27***
Oc.S/BS (%)	0.85 ± 0.47***	1.76 ± 0.47	0.96 ± 0.27***
Calcitonin (pg/ml)	63 ± 5***, ^$$$^	44 ± 6	47 ± 10
Exendin-4 (pg/ml)	ND	ND	ND
CTx-I (ng/ml)	7.4 ± 1.8***	13.7 ± 4.4	8.5 ± 1.7**
P1NP (ng/ml)	21.0 ± 3.6**, ^$$^	27.8 ± 4.0	28.0 ± 2.2

One way ANOVA with Tukey’s multiple comparisons test.

** p<0.01

*** p<0.001 vs. OVX+Vehicle;

$$ p<0.01

$$$ p<0.001 vs. OVX+Ex-4; ND: not detectable.

**Table 3:** Bone histomorphometry at the proximal tibia metaphysis and plasma levels of calcitonin after subcutaneous administration of Ex-4-BSA.

	Sham + Vehicle (n=8)	OVX + Vehicle (n=8)	OVX + Ex-4-BSA (n=8)
O.Th (μm)	6.6 ± 1.8	5.9 ± 1.7	5.9 ± 1.4
OS/BS (%)	3.5 ± 2.1	3.8 ± 2.3	3.8 ± 2.1
MAR (μm/day)	2.78 ± 0.51	2.56 ± 3.37	2.51 ± 0.46
MS/BS (%)	14.1 ± 1.4	14.6 ± 1.1	14.0 ± 1.0
BFR/BS (μm^3^/μm^2^/day)	0.389 ± 0.069	0.375 ± 0.072	0.350 ± 0.058
N.Oc/B.Pm (mm^-1^)	1.11 ± 0.30***, ^$$$^	2.05 ± 0.34	1.97 ± 0.24
Oc.S/BS (%)	0.96 ± 0.17***, ^$$$^	2.12 ± 0.35	2.05 ± 0.19
Calcitonin (pg/ml)	64 ± 5***, ^$$$^	42 ± 7	106 ± 15***
CTx-I (ng/ml)	8.6 ± 0.7***, ^$$$^	16.4 ± 2.6	16.4 ± 2.2
P1NP (ng/ml)	19.7 ± 2.0*, ^$^	26.8 ± 7.7	26.5 ± 3.7

One way ANOVA with Tukey’s multiple comparisons test.

* p<0.05

*** p<0.001 vs. OVX+Vehicle;

$ p<0.05

$$$ p<0.001 vs. OVX+Ex-4-BSA

## Data Availability

The data that support the findings of this study are available from the corresponding author upon reasonable request.

## Lay Summary

Previous work found that the hormone GLP-1 is essential for strong bones, but how it helps was unclear. This study discovered that medications mimicking GLP-1, like exendin-4, improve bone strength and structure in mice, including better bone volume and collagen quality. Interestingly, exendin-4’s effects were observed when delivered to the brain but not when prevented from reaching it. This suggests GLP-1 influences bones through brain signals rather than acting directly on bone. While GLP-1 treatments show promise for preventing bone weakness, more research is needed to understand this brain-bone connection.

## New & Noteworthy

This study discovered that medications mimicking GLP-1, like exendin-4, improve bone strength and structure in mice, including better bone volume and collagen quality. Interestingly, exendin-4’s effects were observed when delivered to the brain but not when prevented from reaching it. This suggests GLP-1 influences bones through brain signals rather than acting directly on bone. While GLP-1 treatments show promise for preventing bone weakness, more research is needed to understand this brain-bone connection.
