# Supplementary material for: The GLP-1 analogue, exendin-4, improves bone material properties and strength through a central relay in ovariectomized mice
Source: Am J Physiol Endocrinol Metab. Author manuscript; Available in PMC 2025 Oct 1. (PMC7618104; doi:10.1152/ajpendo.00086.2025)

Supplementary Figure S1: Schematic design of the mouse model for ovariectomy-induced bone fragility. Three separate studies were conducted, each with the same temporal design and skeletal assessments. In Study 1, saline or exendin-4 was administered subcutaneously using osmotic mini-pumps. In Study 2, saline or exendin-4 was administered via intracerebroventricular infusion through an osmotic mini-pump connected to a brain infusion kit, which was inserted into the lateral ventricle. In Study 3, saline or exendin-4-BSA was administered subcutaneously using osmotic mini-pumps.

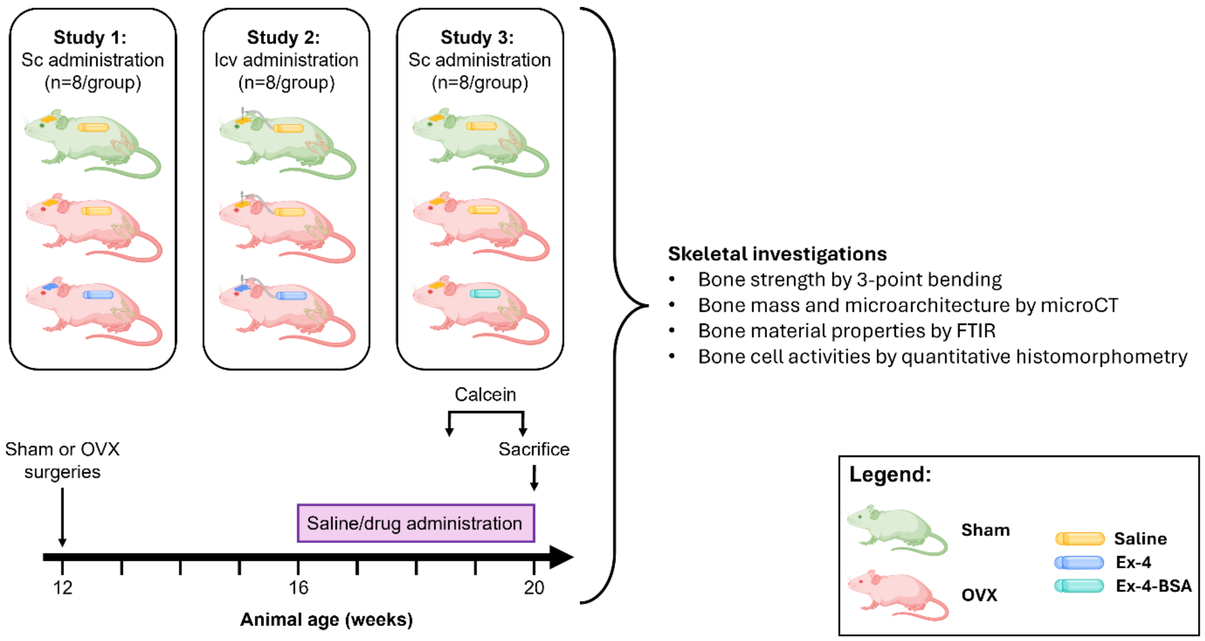

Supplementary Figure S2: Effect of Exendin-4 on body mass. Mouse were treated with either vehicle or Exendin-4 (Ex-4) administered for 4 weeks by subcutaneous (Study 1) or intracerebroventricular (Study 2) administration. In Study 3, Ex-4-BSA was administered subcutaneously for 4 weeks. No significant differences were observed between vehicle- and Ex-4–treated OVX animals. Statistical analysis was performed using one-way ANOVA with Tukey's multiple comparisons test. ***: p<0.001 vs. Sham+Vehicle.

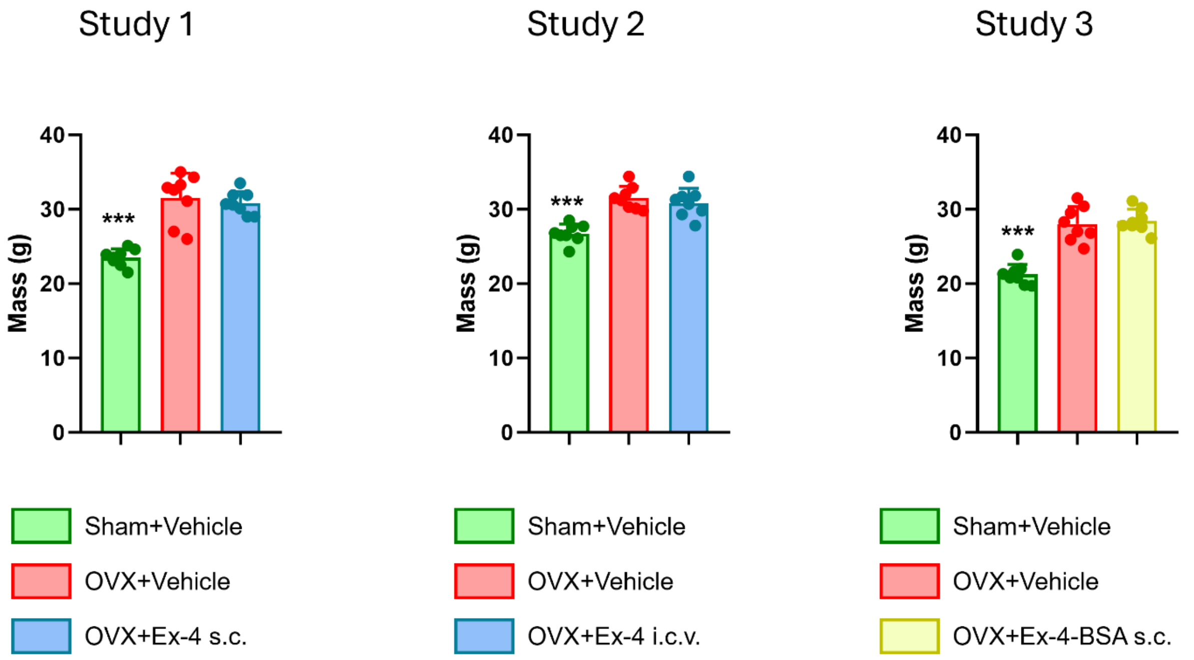

Supplementary Figure S3: Effect of Exendin-4 on osteogenic differentiation of bone marrow stromal cells (BMMSCs). BMMSCs were cultured under osteogenic conditions and treated with either vehicle or Exendin-4 (Ex-4) for the duration of the differentiation protocol. Parathyroid hormone at 50 nM was used as positive control. (A) Alkaline phosphatase (ALP) activity was assessed at day 14 as an early marker of osteoblast differentiation. (B) Alizarin Red S (ARS) staining was performed at day 21 to evaluate extracellular matrix mineralization as a late marker of osteogenesis. Quantification of ALP and mineralization signals is shown as mean ± SD (n = 5 independent experiments). No significant differences were observed between vehicle- and Ex-4–treated cells. Statistical analysis was performed using one-way ANOVA with Tukey's multiple comparisons test. ***: p<0.001 and *: p<0.05 vs. Vehicle.

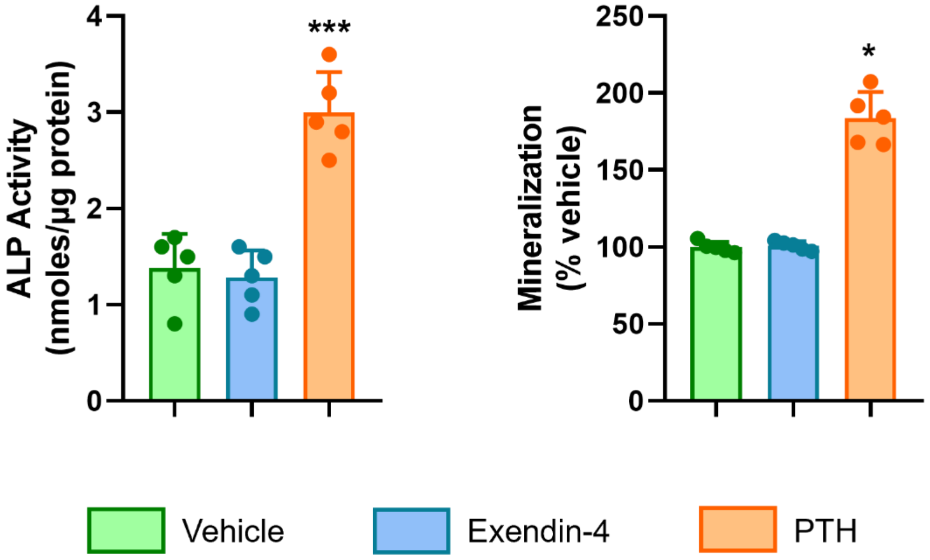

Supplementary Figure S4 – Effect of Exendin-4 on osteoclast differentiation in vitro. Splenocytes were isolated from BALB/c ovariectomized (OVX) mice at 12 weeks of age, 4 weeks post-ovariectomy, and cultured in osteoclast differentiation medium containing M-CSF and RANKL, with or without 10 nM Exendin-4. Cells were cultured for 7 days, and osteoclast differentiation was assessed by TRAP staining. D-Ala2-GIP was used at 10 nM and served as positive controls. Statistical analysis was performed using one-way ANOVA with Tukey's multiple comparisons test. ***: p<0.001 vs. M-CSF+RANKL+Vehicle.

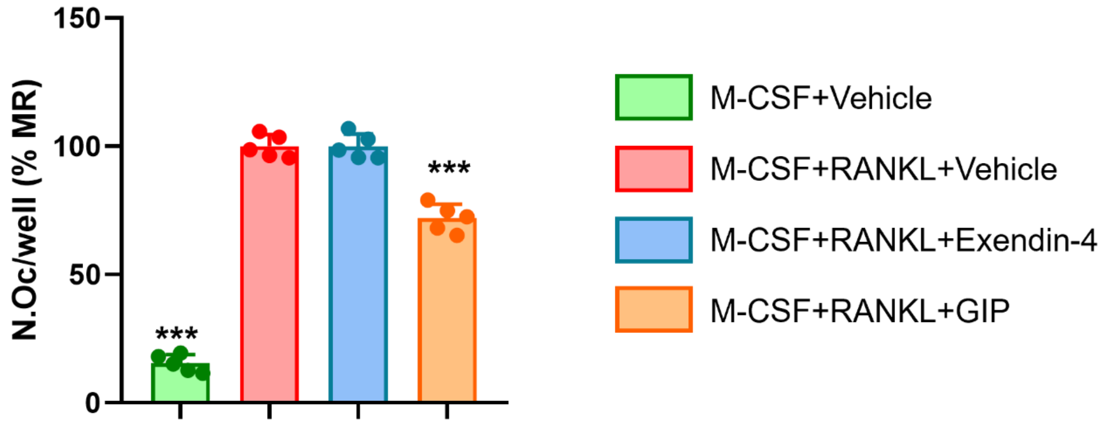

Supplement: Supplementary Figures [file EMS207942-supplement-Supplementary_Figures.pdf]
